# Evaluation of Fourier transform infrared spectroscopy as a first-line surveillance typing tool for *Serratia* spp. isolates derived from clinical materials

**DOI:** 10.3389/frabi.2026.1781370

**Published:** 2026-07-23

**Authors:** Tristan Lüdecke, Klaus-Peter Hunfeld

**Affiliations:** 1 Institute for Laboratory Medicine, Microbiology and Infection Control, Northwest Medical Center, Academic Teaching Hospital, Medical Faculty, University of Frankfurt/Main, Frankfurt am Main, Germany; 2 Society for the Promotion of Quality Assurance in Medical Laboratories (INSTAND), e.V., Düsseldorf, Germany

**Keywords:** bacterial typing, epidemiology, Fourier transform infrared spectroscopy, outbreak, public health, *Serratia*, whole genome sequencing

## Abstract

**Introduction:**

Fourier transform infrared spectroscopy (FTIR) has been evaluated as a typing method for a number of pathogens; however, very little research has been conducted on the genus Serratia.

**Methods:**

We examined whether FTIR was able to correctly cluster clinical isolates of Serratia to a comparable degree as whole genome sequencing (WGS) coupled with a subsequent bioinformatic comparison, the gold standard for determining phylogenetic relationships in epidemiology. FTIR was performed with the IR Biotyper (Bruker, Bremen, Germany) using the standard settings. Over a period of six weeks, 28 Serratia isolates found in clinical and environmental hygiene samples investigated in our laboratory were collected and preserved. Retrospective strain typing was performed using both methods, and the results were subsequently compared.

**Results:**

Our study showed a PPV of 0.842 for the correct identification of closely related isolates and an NPV of 1.0 for the correct identification of unrelated isolates. Concordance was measured using the Adjusted Rand index (AR) and the Adjusted Wallace coefficient (AW), with WGS and subsequent bioinformatic analysis as the reference method. This resulted in an AR of 0.742.

**Discussion:**

Our findings indicate, although with some limitations, the utility of using the IR Biotyper as a rapid and cost-effective first-line screening tool for investigating the epidemiological relatedness of Serratia spp. in a clinical microbiology laboratory when an outbreak is suspected. Consequently, the FTIR method may provide a complementary analytical addition to the arsenals of clinical microbiology laboratories that carry out epidemiological surveillance to aid infection control in clinical settings.

## 1 Introduction

Nosocomial infections pose a potential risk to patients within modern healthcare systems. The primary complication and concern is a significant increase in mortality (WHO, 2011). However, even in the absence of life-threatening complications, a rise in healthcare costs due to a prolonged hospital stay and the increased use of antibiotics constitute a complicating risk factor. According to the World Health Organization (WHO), the rate of nosocomial infections in hospitalized patients is 7.6% in the developed world. Patients requiring treatment in intensive care units are at an even greater risk, with a reported incidence of infection there of 30% (WHO, 2011).

*S. marcescens*, the type strain of the genus *Serratia*, is a facultative human pathogen belonging to the order Enterobacterales. It is primarily encountered as a causative agent of nosocomial infections, particularly in neonatal intensive care units, with sink drains as one of the main reservoirs (Bourdin et al., 2023). Nevertheless, *Serratia* can colonize a range of wet surfaces including artificial nails, as well as medical equipment, medical vials, the human gastrointestinal and respiratory tract, and materials such as lotions, antiseptics, and even quaternary ammonium-based disinfectants when handled incorrectly (Hanczvikkel et al., 2024). Moreover, there have been reports of multi-year-long outbreaks (Kamali et al., 2024) as well as nationwide clonal spreading of *Serratia* spp., further emphasizing its significance as a facultative pathogen in humans (Taxt et al., 2025).

In recent years, several new *Serratia* spp. such as *S. sarumanii* (Klages et al., 2024) and *S. montepellierensis* in 2024 (Blackburn et al., 2024), and *S. nevei*, and *S. bockelmannii* in 2020 (Cho et al., 2020) have been discovered. However, this is currently not reflected in clinical practice since *Serratia* spp. are rarely identified by methods other than the most common one: MALDI-TOF. This method, however, is currently unable to reliably differentiate between *Serratia* spp. as there is a high degree of similarity between the mass spectra, and some of the recently discovered species have not yet been included in the databases (Harch et al., 2025).

As only 1-2% of nosocomial infections are caused by *S. marcescens* (Khanna, 2013), an epidemiological increase in the isolation of *Serratia* spp. – as observed in the context of our investigation – should prompt further epidemiological workup to prevent further possible spread of the pathogen. The bacterium, like other Enterobacterales, presents a clinical threat to patient safety as it can cause a variety of complications depending on the site of infection. Another key pathogenic factor is the potential presence of beta-lactamases such as AmpC (ampicillin-resistant gene group C), ESBL (extended-spectrum beta-lactamase), and carbapenemases such as NDM and KPC (Vanni et al., 2023). These can render a range of antibiotics ineffective and thus potentially complicate empirical antibiotic treatment.

To effectively control outbreaks of pathogens like *Serratia* spp. in the clinical setting, it is beneficial to quickly identify clonality of isolates, possible transmission routes, and reservoirs so that appropriate countermeasures can be rapidly implemented (Brouqui et al., 2017). Such investigations were traditionally carried out using labor-intensive clinical epidemiological methods, such as line lists and two-by-two tables. These investigations were then further confirmed through pulsed-field gel electrophoresis (PFGE) which was long considered the gold standard in epidemiological outbreak analysis (Peacock et al., 2002). This diagnostic strategy has changed in recent years since the breakthrough of next generation sequencing (NGS) as a way to facilitate WGS coupled with subsequent bioinformatic analysis – a new and very reliable method for epidemiological strain typing. Today this approach is considered the “new” gold standard for determining the epidemiological and phylogenetic relationship of clinical isolates due to its significantly higher accuracy and better availability at increasingly affordable analytical costs (Bertelli and Greub, 2013; ECDC, 2018; Peacock et al., 2018). However, WGS and the subsequent need for complex bioinformatic analysis require powerful computing resources as well as qualified personnel with expertise in the processing and interpretation of the detected sequences (Bagger et al., 2024). As a result, establishing such technologies on a local level can be non-feasible for many resource-constrained hospital laboratories. Furthermore, preparing and executing the process remains time-consuming despite the many groundbreaking innovations in the field of NGS, and this leads to significant delays in the delivery of information. Consequently, there is a strong need for a simpler and faster way of identifying epidemiological relatedness under routine conditions as part of the clinical surveillance of isolates detected in clinical materials by routine microbiology laboratories. This would enable these laboratories to prevent and contain possible nosocomial outbreaks in the course of their clinical infection control services (Berbel Caban et al., 2020).

FTIR is a method that analyzes the interferograms generated when infrared light is passed through a sample. In analytical chemistry, a recorded spectrum can be compared with a spectral database to identify pure substances (Griffiths, 1983). Microorganisms can also be studied and differentiated using this technology as the resulting spectrum is determined by the organism’s molecular makeup (Naumann et al., 1991). Different substance classes contribute to the absorption bands to varying degrees. A bacterium’s FTIR spectrum is therefore a complex mixture of information about the chemical composition of the analyzed sample. FTIR spectroscopy is a promising method for investigating suspected outbreak situations because it can be rapidly employed and is relatively cost efficient. Since 2017, Bruker has commercially offered an integrated system for bacterial subtyping based on FTIR technology. To date, only one study has evaluated this method in connection with the epidemiological analysis of clinical *Serratia* spp. isolates. However, the isolates included in this investigation turned out to be largely unrelated, preventing any significant conclusions to be drawn regarding FTIR performance when testing *Serratia* spp. isolates (Uribe et al., 2023).

In contrast to previous studies, the primary aim of our study was to evaluate the performance of FTIR using the IR Biotyper as a rapid initial typing method to screen for potentially closely related *Serratia* isolates (n=26) under routine conditions, and to compare its performance in the clinical setting against WGS as the reference method. To date, our assortment of *Serratia* spp. isolates is the largest collection to be comprehensively studied in this regard.

## 2 Materials and methods

### 2.1 Study design

A retrospective study was conducted in which clinical *Serratia* spp. isolates were recovered between February and March 2024 during a 6-week period of increased incidence of this organism from adult ventilated patients treated in a neurological intensive care unit at one of the collaborating clinical centers, that received routine microbiological testing services from our microbiology department. The isolates uniformly displayed *Serratia* spp.-typical antimicrobial susceptibility testing results (including some AmpC ß-lactamase overexpression), but without phenotypic or molecular evidence for multidrug resistance. To investigate the possible outbreak, 24 first isolates from respiratory materials (tracheal secretions, bronchoalveolar lavage, pharyngeal swabs) were included in the study as well as a pair of isolates derived from the environment of this institution (18, 19), and two reference isolates obtained from two epidemiologically unrelated cooperating institutions (24, 26). The final set of strains therefore consisted of 28 *Serratia* isolates. The isolates were pseudonymised by assigning consecutive numbers to ensure patient anonymity. All isolates were extracted from the routine diagnostic testing performed on clinical materials or environmental samples in our laboratory. No additional sample collection apart from the routine diagnostic processing of samples was performed. Therefore, obtaining informed written consent or approval from an ethics committee was not required.

### 2.2 Isolates and culture conditions

All isolates were cultured on conventional sample-specific culture media (MacConkey-Agar Becton Dickinson GmbH, Heidelberg, Germany) and identified using the MicroScan WalkAway plus system (Beckman Coulter, Brea, United States) as part of routine susceptibility testing carried out in accordance with the CLSI guidelines (Clinical & Laboratory Standards Institute, M100, 2024). Following microbiological testing, all isolates were stored at -20 °C using a Microbank™ sample container (Pro-Lab Diagnostics™, Ontario, Canada). After the collection period, the isolates were re-inoculated on Columbia agar with 5% sheep blood (Becton Dickinson GmbH, Heidelberg, Germany) using the streak plate method. Incubation was performed for 24 hours at 37 °C and a single colony was transferred to a new Columbia agar plate with 5% sheep blood in order to facilitate confluent growth. This was then incubated for another 24 hours at 37 °C. DNA extraction and preparations for FTIR were subsequently carried out. Matrix-assisted laser desorption ionization-time of flight mass spectrometry (MALDI-TOF MS, Bruker Daltonik GmbH, Bremen, Germany) was performed to ensure correct species identification.

### 2.3 Fourier transform infrared spectroscopy

#### 2.3.1 IR Biotyper application

Sample preparation for the IR Biotyper was carried out according to the manufacturer’s instructions. Initially, 50 µl of 70% ethanol was added to a Bruker suspension vial containing metal cylinders (part of the IR Biotyper Kit Part No: 1851760). This was followed by the addition of an adequate amount of the previously subcultivated isolate using a 1 µl inoculation loop. A homogeneous suspension was prepared with a vortex shaker (Heidolph REAX 1 DR, Heidolph, Schwabach, Germany). To increase the surface tension, 50 µl of distilled water was added. Five technical replicates were prepared per isolate. Afterwards, 15 µl of the prepared suspension was applied to each of the five targets on the silicon target plate (Part No. I23258P, Bruker, Germany) and left at room temperature until completely dry. During the drying process, isolates and the positions of the technical replicates were entered into the IR Biotyper software (OPUS 8.2.28). Next, the silicon target was placed in the corresponding frame, and the FTIR spectra acquisition was initiated. The isolates were measured using the standard settings according to the manufacturer’s instructions in a wave number range of 800–1300 cm^-^¹ since this region tends to show the best performance in terms of classification (Candela et al., 2025). This procedure was repeated 3 times for each isolate to obtain at least 15 infrared frequency spectra of high analytical quality. Each set of spectra was obtained from freshly prepared subcultures.

The IR Biotyper software (v. 4.0.6.7365; Bruker GmbH, Bremen, Germany) and the OPUS software (v. 8.2.28; Bruker GmbH) were used to conduct complex, multi-stage data analysis on an automated basis to extract, process, and visualize the relevant information contained in the respective IR spectra (Quintelas et al., 2018; Burckhardt et al., 2019; Martak et al., 2019; Novais et al., 2019; Rakovitsky et al., 2020). First, the raw data of an IR spectrum was modified by applying baseline corrections, smoothing, and normalization. This ensured that differences in absorption were attributable to biological variations rather than possible experimental fluctuations (Shen et al., 2018). A calculation of the second derivative, vector normalization, and spectral quality control were performed by the OPUS software using the standard settings. The IR Biotyper software was used to create a project to which all acquired FTIR spectra that passed the quality checks were added.

Hierarchical cluster analysis was performed based on the Euclidean distance using the UPGMA algorithm (unweighted pair group method with arithmetic mean). This is an agglomerative method that successively merges the two closest clusters, thereby generating a dendrogram. Ultimately, the software automatically creates a cut-off based on the Simpson’s Index of Diversity to group the isolates (Hunter and Gaston, 1988).

The manufacturer recommends adding unrelated isolates to a study set to help assess phylogenetic relationships. Therefore, we added two unrelated *Serratia* spp. isolates from two different sources to the isolates of interest. These isolates (24 and 26) were confirmed to be unrelated by cgMLST and were thus excluded in the final evaluation that compared the performance of FTIR and cgMLST.

Since no optimized cut-off for *Serratia* spp. had yet been defined, the isolates were grouped based on the automatically generated cut-off calculated by the IR Biotyper software (v. 4.0.6.7365; Bruker GmbH, Bremen, Germany) and the OPUS software (v. 8.2.28; Bruker GmbH, Bremen, Germany). Isolates with a spectral distance value corresponding to or below the automatically generated cut-off were considered to belong to the same FTIR cluster, while isolate pairs not meeting this criterion were considered to belong to different FTIR clusters. When interpreting the relatedness of the acquired spectra, the dendrograms generated from the averaged spectra as well as individual spectra were evaluated together. The averaged spectrum of each isolate was used to create a dendrogram for determining preliminary clusters. Then, in a second step, a dendrogram was calculated from all individual spectra. Isolates presenting as singletons were regarded as unrelated and eliminated from the preliminary clusters (2, 9, 12, 17, 23, 25). This resulted in the final FTIR-clusters depicted in **Table 1**. Three isolates were entirely removed from the performance evaluation. Isolates 24 and 26 had only served as reference spectra, and isolate 10 was disregarded as it was contaminated with *Stenotrophomonas maltiphilia*, which was brought to light during the bioinformatic analysis.

**Table 1:** Isolate overview listing: isolate ID, cgMLST cluster, FTIR cluster, species determination with TYGS.

ID	cgMLST	FTIR	TYGS
5	I	A	SEUR
8	I	A	SEUR
13	I	A	SEUR
15	I	A	SEUR
27	I	A	SEUR
28	I	A	SEUR
6	II	B	SEBO
16	II	B	SEBO
7	III	C	SEBO
14	III	C	SEBO
22	III	C	SEBO
1	IV	D	SESA
4	IV	D	SESA
11	IV	D	SESA
18	V	E	SEMA
19	V	E	SEMA
2			SENE
3		D	SESA
9			SENE
10		X	SEUR
12			SESA
17			SEUR
20		D	SESA
21		A	SEUR
23			SENE
24		X	SESA
25			SESA
26		X	SESA

Isolate IDs are written in bold numbers in all tables for improved visibility and to differentiate them from other numeric values. Roman numerals are assigned to cgMLST clusters. Singletons are left blank. Letters were assigned to FTIR clusters. Singletons are left blank. Excluded isolates: the reference isolates 24, 26 and contaminated sample 10 are marked with an X in the FTIR column and were not included in the evaluation. Serratia spp. SEUR: Serratia ureilytica, SEBO: Serratia bockelmanii, SESA: Serraia sarumanii, SEMA: Serratia marcescens, SENE: Serratia nevei.

### 2.4 Whole genome sequencing

#### 2.4.1 DNA extraction and WGS

Nucleic acids were extracted using the MagNA Pure 24 System (Roche Diagnostics, Mannheim, Germany) and the Pathogen 1000 3.2 protocol. Following extraction, the samples were treated with 4 µl of RNase (100 mg/ml) (QIAGEN^®^, Venlo, Netherlands) for 2 minutes to degrade any RNA that could interfere with next generation sequencing (NGS). This was followed by column purification using the High Pure PCR Template Preparation Kit (Roche Diagnostics, Mannheim, Germany). Finally, the DNA was quantified using the Qubit (Thermo Fisher Scientific, Waltham, USA). NGS was conducted on the extracted, purified, and quantified DNA on a NovaSeq X+ platform using the NovaSeq X Series Reagent Kit (Illumina, San Diego, USA). The NEBNext Ultra II FS DNA LibPrep Kit (New England Biolabs) was employed to create shotgun libraries for all samples. Sequencing was performed using a 2x150 bp paired-end workflow.

#### 2.4.2 Bioinformatics

A quality check was performed using FastQC (version 0.12.0) on standard settings, followed by adapter trimming with Trimmomatic (version 0.40) on the recommended default settings (seed mismatches = 2, palindrome clip threshold = 30, and simple clip threshold = 10). The reads were subsequently down-sampled in SKESA (version 2.4.0) on default settings to an average coverage of 180× (based on NZ_HG326223.1 as seed genome), followed by *de novo* assembly using SKESA on default settings. Assembly post-processing and polishing were carried out with BWA-MEM (version 0.7.15, parameters: mem, paired end, consensus caller: no coverage minimum required consensus call read support 60.0%). A contamination check was performed with Mash Screen (version 2.3) using default thresholds (Identity >=0.95, Shared-hashes >=100) with the ‘Winner takes all’ option enabled to remove redundancy in the results.

##### 2.4.2.1 cgMLST

Ridom SeqSphere+ was used to screen the assemblies for the alleles defined in the cgMLST scheme for *Serratia marcescens*. This was developed and curated by Kampmeier et al. and comprises 2,692 alleles. According to the scheme, isolates differing by less than or equal to 12 alleles are considered to belong to the same cluster. This suggests strain transmission if there are also temporal and local epidemiological data that support these findings. Isolates differing by more than 12 alleles are assumed to be unrelated (Kampmeier et al., 2022).

##### 2.4.2.2 Cluster-based SNP distance

Raw sequencing reads were pre-processed with BBDuk (version 38.84) for adapter trimming and quality trimming to remove bases below a Phred score of 30 from both ends. The resulting trimmed reads were mapped against a previously assembled representative genome from the respective cluster using the Geneious Mapper on medium sensitivity/fast settings. Single nucleotide polymorphism (SNP) calling was performed with Geneious Prime (version 2025.2.2) using the “find variations/SNPs” workflow with a minimum coverage of 10x, a minimum variant frequency of 95%, a maximum variant *p*-value of 10^-6^, and a minimum strand-bias *p*-value of 10^5^.

#### 2.4.3 Species determination

Species determination during the routine diagnostic workup was carried out using the biochemical tests integrated into the antibiotic susceptibility testing with the MicroScan WalkAway plus system (Beckman Coulter, Brea, United States). MALDI-TOF was performed to further solidify the species identification. WGS was followed by several analytical methods, namely: MASH Screen (MinHash (Ondov et al., 2016) [min-wise independent permutations locality sensitive hashing scheme (Broder, 1998)]), ANI (average nucleotide identity) (Konstantinidis and Tiedje, 2005) and TYGS (type strain genome server) (Meier-Kolthoff and Göker, 2019). TYGS is commonly regarded as the most accurate method for identifying species and was used by us to define the species of an isolate.

### 2.5 Statistical analysis

The congruence of FTIR with cgMLST was assessed using the Adjusted Rand index (AR) and the Adjusted Wallace coefficient (AW) with 95% confidence intervals (CI). AR compares the overall congruence between two typing methods (Santos and Embrechts, 2009) while AW compares the agreement of two typing methods, assigning one of them as the reference method (Severiano et al., 2011). AR and AW values range from 0 to 1, where 0 means agreement by chance and 1 means optimal correlation between both methods (Severiano et al., 2011; Hoffman et al., 2015). To determine the sensitivity and specificity of FTIR in the detection of related isolates, a true positive was defined as an isolate which was classified by cgMLST as closely related (≤ 12 allele difference) and grouped into a cluster by the IR Biotyper. A false positive was considered to be an isolate that was classified by cgMLST as not closely related (> 12 allele difference) but grouped into a cluster by FTIR. A true negative was defined as an isolate considered by cgMLST as being not closely related (> 12 allele difference) and not grouped into a cluster by FTIR. A false negative was specified as an isolate classified by cgMLST as being closely related (≤ 12 allele difference) but not grouped into a cluster by FTIR (Kampmeier et al., 2022).

## 3 Results

### Identification of isolates at the subspecies level

The MicroScan WalkAway plus system (Beckman Coulter, Brea, United States) reported *S. marcescens* for all isolates. MALDI-TOF similarly reported *S. marcescens* for most isolates and *S. nematodiphilia* for 5 isolates. The NGS-derived results of MASH Screen and TYGS revealed the heterogeneous makeup of the studied isolates. This consisted of several species including *S. ureilytica, S. bockelmannii, S. sarumanii, S. marcescens* and *Serratia nevei.* Furthermore, the species identification unveiled a potentially new *Serratia* sp. which is currently under investigation. The results are displayed in **Table 1**.

### 3.1 cgMLST analysis

**Figure 1:**
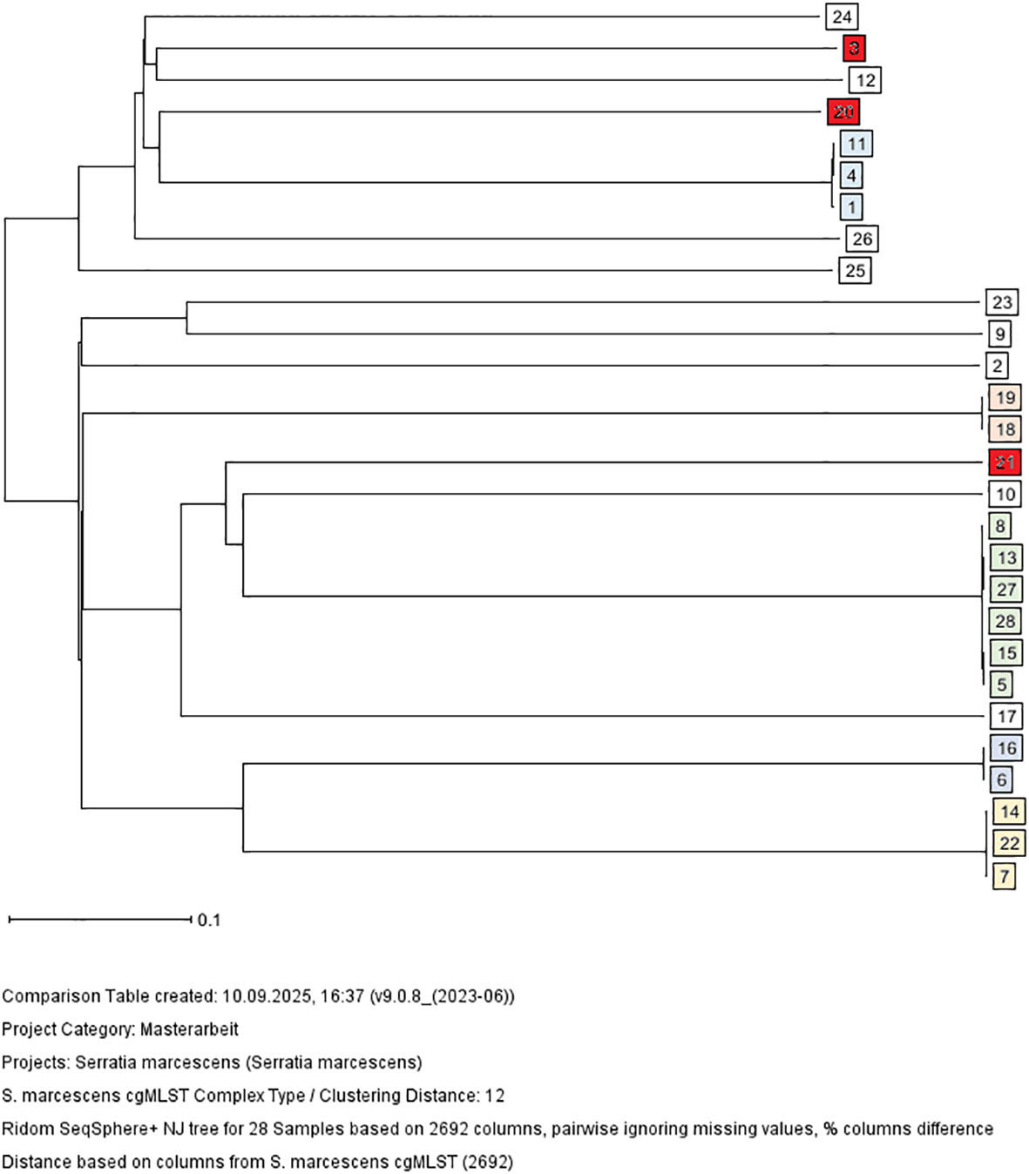
cgMLST dendrogram derived from the cgMLST results using neighbor joining. Color coding according to **Table 1**. False positive FTIR results are highlighted in red.

The cgMLST analysis of the NGS data detected five clusters in the study set (see **Figure 1**). Roman numerals were assigned to the cgMLST clusters. Of the 25 isolates studied, 16 were part of a cluster and 9 isolates were singletons. A total of 14 different strain types were identified. One sample (no. 10) was excluded from the evaluation as it was contaminated with *Stenotrophomonas maltophilia*.

### 3.2 Pairwise SNP distance calculation

Pairwise SNP distances between the isolates inside a given cgMLST cluster were calculated to further enhance the certainty of epidemiological linkage inside the cgMLST clusters. The SNP distance ranged from 0–3 in clusters I-IV, indicating clonal relationships between the isolates in those clusters. The two isolates making up Cluster V, on the other hand, presented a pairwise SNP distance of 30. Despite the relatively high SNP distance between the two samples, the isolates were considered epidemiologically linked given that samples 18 and 19 were recovered from the drain and the trap of the same hospital sink.

### 3.3 Epidemiological typing using FTIR

**Figure 2:**
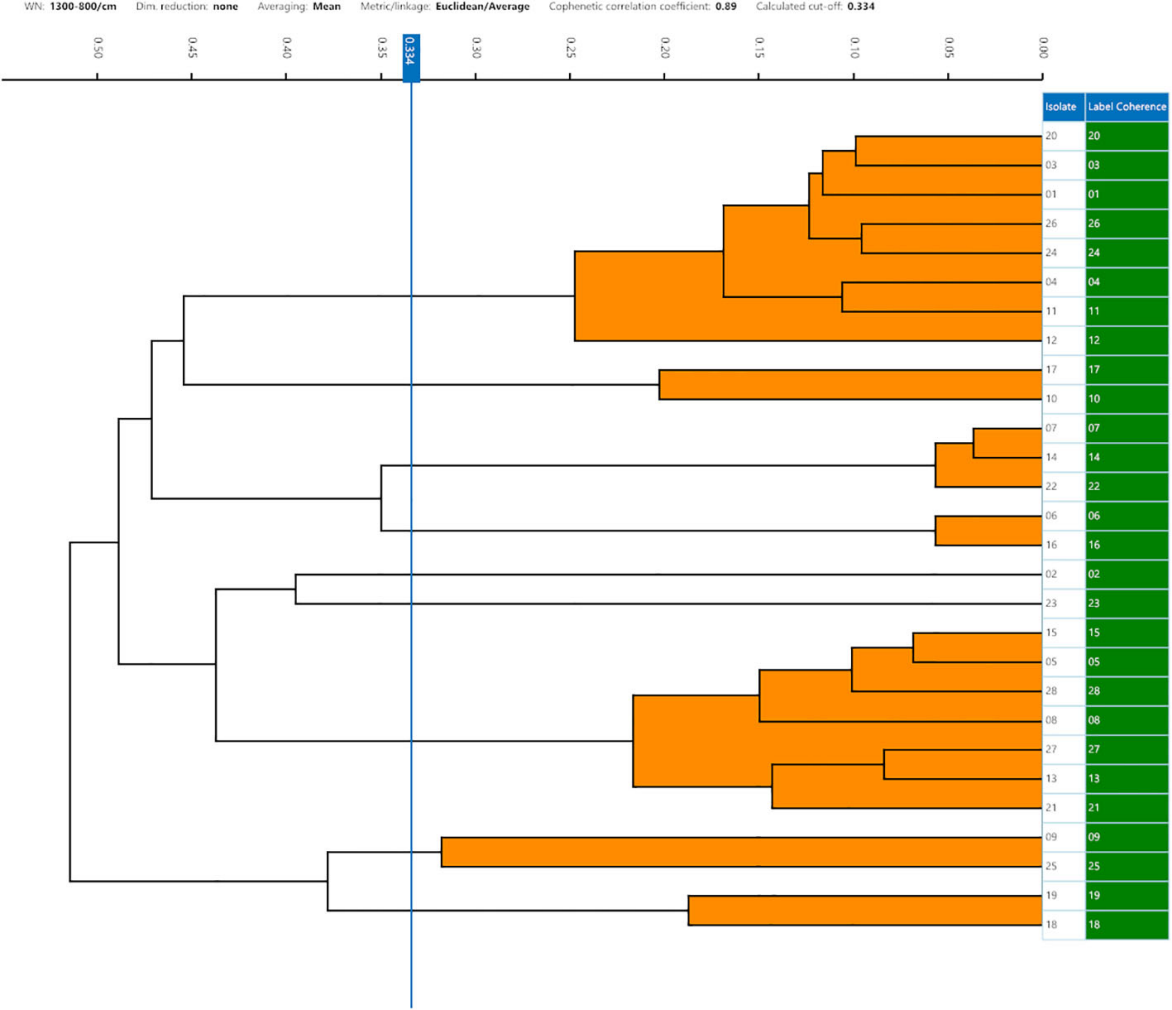
Dendrogram obtained by clustering the averaged FTIR spectra of the 28 isolates of *Serratia* spp. Settings = exploration method: Euclidian average; cluster quality criterion: isolate; cut-off optimization: optimized silhouette coefficient.

The preliminary clusters were derived from the averaged spectra dendrogram (see **Figure 2**).

As outlined above, using the dendrogram generated from all FTIR spectra, isolates 2, 9, 12, 17, 23 and 25 were identified as singletons and therefore removed from the preliminary FTIR clusters. The reference isolates (24, 26) and contaminated sample 10 were entirely removed from the evaluation. This resulted in the final FTIR clusters listed in **Table 1**.

The 25 potentially epidemiologically linked *Serratia* isolates included in the final evaluation were grouped by the IR Biotyper into 5 clusters consisting of 19 isolates and 6 singletons. The two largest clusters were cluster A (*n* = 7) and cluster D (*n* = 5). In order to infer the correct and incorrect attribution of an isolate to a cluster, the result of the cgMLST analysis was defined as true to serve as a reference. Of the 5 FTIR clusters, two were not fully concordant with cgMLST, showing the inclusion of additional, unrelated isolates (cluster I/A n=1; IV/D n=2). Full agreement was found between FTIR and cgMLST in the remaining three clusters (II/B, III/C and V/E) and 6 singletons (isolates: 2, 9, 12, 17, 23, 25). Simpson’s Index of Diversity (SID) was calculated to compare the discriminatory power of the two methods (see **Table 2**).

**Table 2:** Simpson’s Index of Diversity and the respective 95% confidence intervals calculated using large sample approximation.

Method	# of partitions	SID	CI (95%)
FTIR	10	0.88	(0.804-0.956)
cgMLST	14	0.923	(0.861-0.985)

An AR of 0.742 (0.456-1.000) and an AW of 0.590 (0.315-0.865) were calculated using an online tool (Comparing Partitions Website, 2011).

Of the 25 clinical isolates investigated, FTIR correctly identified 16 as epidemiologically linked. Using our approach, not a single closely related isolate was deemed unrelated by the IR Biotyper. Nevertheless 3 isolates were falsely assigned to a cluster when they were in fact unrelated, as shown by a cgMLST analysis. The remaining 6 isolates were correctly identified as not being epidemiologically linked (see **Table 3**).

**Table 3:** FTIR – cgMLST contingency.

Test result	cgMLST pos	cgMLST neg
FTIR pos	16	3
FTIR neg	0	6

These findings amount to a sensitivity of 1.0 in our study population and a specificity of 0.667 (see **Table 4**).

**Table 4:** Performance metrics.

Sensitivity	1.0
Specificity	0.667
Accuracy	0.88
PPV	0.842
NPV	1.0

## 4 Discussion

In this study, an episodic increase in *Serratia* spp. isolates, which were cultivated from clinical materials originating from a collaborating clinical center for ventilated patients, suggested a possible outbreak situation. FTIR is known to be able to adequately determine epidemiological relatedness in clinically isolated strains for a number of pathogens, including Gram-negative bacteria (Lombardo et al., 2021) (Vogt et al., 2019), (Martak et al., 2019), (Dinkelacker et al., 2018), (Pascale et al., 2022), as well as Gram-positive bacteria (Teng et al., 2022), non-tuberculous mycobacteria (Bisognin et al., 2022), and yeasts (Cardona et al., 2012). Thus, FTIR could serve as a screening test in clinical settings to rapidly identify an epidemiological linkage when an outbreak is suspected.

In our study, an epidemiological linkage between isolates was successfully identified in all cases (sensitivity 1.0). The findings of our study also tend to strengthen the evidence that rapid initial strain typing can be performed for *Serratia* spp. under clinical microbiological conditions using automatically generated cut-offs on the IR Biotyper. This was also demonstrated in a recent study on a single *S. marcescens* cluster containing two related isolates (Uribe et al., 2023). However, in our investigation, incorrect grouping of unrelated isolates also occurred in 3 cases – a finding that significantly differs from previous results. Likewise, a specificity of 0.667 is not ideal but might be expected for a screening test based on phenotypic traits and performed under routine laboratory conditions. It clearly indicates that additional evaluations are necessary to further increase FTIR specificity for epidemiological clustering of *Serratia* spp. The overall performance of FTIR for typing nosocomial pathogens using the IR Biotyper showed a PPV of 55.81% and a NPV of 86.79% (Uribe et al., 2023). A recently published meta-analysis of 149 studies found an overall accuracy of 78% for Enterobacterales (Al-Fraihat et al., 2025). In comparison, the correct typing of epidemiological relatedness in clinical, outbreak-related *Serratia* isolates, as found in our study, was noticeably elevated, with a PPV of 84%, an NPV of 100%, and an overall accuracy of 88%, suggesting better-than-average performance with respect to Enterobacterales as well as nosocomial pathogens as a whole.

Previous studies have argued that FTIR should meet certain criteria before it can be deemed sufficiently congruent with the reference method and be used as an initial screening test in the assessment of strain relatedness. These include a WGS→FTIR AW of 0.95 or higher, which translates to a clustering of nearly all isolates clustered by cgMLST, and an FTIR→WGS AW of 0.5 or higher, which translates to an acceptable amount of false positive results (Teng et al., 2022). These metrics were achieved in our study with a WGS→FTIR AW of 1.0 (95% CI: 1.0-1.0) and an FTIR→WGS AW of 0.590 (95% CI: 0.315-0.865). This indicates an adequate level of performance and a suitability to be used as a first-line screening tool in suspected outbreaks. Nevertheless, the large confidence intervals have to be taken into consideration, strengthening the need for future research.

For comparative purposes, we performed strain typing based on the WGS data through cgMLST in addition to FTIR. Due to the lack of better options, we employed a bioinformatics pipeline developed on *S. marcescens*, even though only isolates 18 and 19 actually belonged to this species. It is important to note, though, that there is no universal genomic cut-off value to determine whether different isolates belong to an outbreak. For cgMLST, a distance of less than or equal to 12 alleles in the pairwise comparison between isolates has been suggested for outbreak delimitation in the case of *S. marcescens* (Kampmeier et al., 2022). Still, this cut-off may not always be correct as genetic variability clearly depends on the time between collection events. Our isolates, however, were collected over a short period of time (6 weeks), and only one first isolate per patient was included, with the exception of samples 18 and 19, which were sampled from the drain and trap of the same sink at the same institution.

An additional phylogenetic investigation, in which SNP distances were calculated pairwise inside the cgMLST clusters, enabled us to confirm the close genomic linkage of the isolates contained in a given cgMLST cluster. Still, the use of SNP cutoffs to determine clonal relationships continues to be a topic of ongoing discussion. In terms of what is considered to be a significant SNP distance, some studies have determined this to be a cut-off of 16 SNPs between epidemiologically linked *S. marcescens* isolates (Chng et al., 2020). Others describe the use of dynamic cut-offs with the temporal dimension of an outbreak in mind (Payne et al., 2021). Nevertheless, the low pairwise distance seen in the majority of clusters (I-IV) leaves no doubt about their close connection. Furthermore, it has been shown that *S. marcescens* isolates sampled from the wound of a patient on the same day can differ by up to 32 SNPs (Ferguson et al., 2023). Therefore, the isolates making up cgMLST Cluster V, which were sampled from a single hospital sink on the same day, were also considered to be epidemiologically linked despite their relatively high SNP distance of 30, emphasizing that there is no universal genomic cut-off value to determine whether isolates belong to an outbreak or not.

In the context of strain typing, the main advantages of FTIR on the IR Biotyper are the relative ease of sample preparation, a faster turnaround time, reduced operational expenditure, and a less resource-intensive data analysis compared to WGS, the gold standard of strain typing. When looking at the occurrence of false positive clustering events, however, the inherent limitations of FTIR as a typing method also come to light. As it is based on the analysis of phenotypic traits rather than genetic similarity, FTIR lacks discriminatory power compared to the purely genetic approach of NGS. Therefore, wrong clustering events can take place and are well-reported in the literature (Muchaamba and Stephan, 2024). The reanalysis of the collected strains with different spectral windows might result in better or worse cluster formation and must be further explored in future research, something that also remains true when exploring other analytical methods.

Further limitations include the need for high consistency when preparing the samples and a strict adherence to the test protocols. This also includes standardized incubation times and a standardized application of the final sample preparation on the silicon microtiter plate. For some genera, an alternative sample preparation method was shown to generate better results over the method proposed by the manufacturer due to the otherwise inhomogeneous bacterial suspension even after extended vortexing (Hu et al., 2021).

Additionally, the type of agar, as well as the incubation time continue to be influencing factors (Wenning et al., 2014; Quintelas et al., 2018), which should be explored further to identify the most suitable growth conditions for an optimal standardized differentiation of *Serratia* spp. It has been shown that certain bacteria can only be investigated with FTIR when grown on certain media, as is the case of *S. aureus*, which has to be grown in liquid media (Becker et al., 2006; Johler et al., 2016). The fact that all isolates in our study originated from one medical center impairs our ability to extrapolate the results of our investigation based on the typing performance and discriminatory power of FTIR in *Serratia* spp. on a more general level. Furthermore, the generalizability regarding the screening of isolates outside of suspected outbreak conditions is somewhat difficult since our samples were collected under the clinical suspicion of an outbreak. This implies a higher pretest probability of relatedness than investigating a more randomly collected set of clinical isolates. As such, analysis of isolates outside typical outbreak conditions could lead to results that diverge from those in our study since larger collections with a high intrinsic diversity are less likely to be assigned as being epidemiologically related than collections consisting of only a handful of isolates (Teng et al., 2022).

The constraints of our study resulting from the limited sample size are reflected in the large 95% confidence intervals of the AR and AW. These were 0.456-1.000 and 0.315-0.865 respectively, thereby ranging from mediocre cluster similarity to near perfect agreement, and from bad predictive reliability to reliable clustering. At the same time, the circumstances of selection correspond to a typical outbreak situation in the clinical setting. As such, our study was not meant to establish and evaluate a classifier to reliably differentiate *Serratia* spp. Instead, our *ad hoc* investigation prompted a chance to gain real-world experience and to investigate the possible suitability of FTIR spectroscopy for the rapid initial screening and typing of *Serratia* spp. strains with regard to their epidemiological relatedness when an outbreak is suspected. Still, our strain collection represents the largest set of *Serratia* spp. isolates studied in this regard to date.

Since our study collective consists of small groups of closely related isolates, with almost every cluster belonging to a different *Serratia* spp., the generalizability of our findings for sample sets consisting of fewer or only one species is limited. Therefore, future studies that incorporate a more systematic evaluation of FTIR in *Serratia* should also investigate one single *Serratia* sp. at a time. Nevertheless, the successful clustering of the individual species, as observed in our study, may indicate the possibility of creating classifiers for a more tailored application when FTIR is used to differentiate *Serratia* spp.

These advantages and limitations indicate that more studies are clearly needed to further evaluate the applicability of FTIR on the IR Biotyper for *Serratia* spp. in clinical practice. Key aspects that need to be addressed in such future studies include investigations of larger sets of isolates sourced from different epidemiological locations, sub-analyses on single-species performance, ring trials on the reproducibility of results when isolates are measured in different laboratories. In addition, an evaluation of various spectral windows and the use of other AI tools for a more sophisticated raw data analysis is needed in order to further improve the robustness and reproducibility of the method in general.

## 5 Conclusion

Our findings build on existing evidence that indicates that FTIR spectroscopy on the IR Biotyper is a rapid, cost-effective, and easy-to-use screening method in suspected outbreak situations (Al-Fraihat et al., 2025) that – with some limitations – is suitable for the rapid initial typing of several *Serratia* spp. derived from clinical materials. However, relying solely on the IR Biotyper for strain typing under the current methodological conditions can also result in misclassifications. From a technical standpoint, the method can be implemented rapidly and performed easily. Indeed, sample preparation only takes a few minutes once the microorganisms have been properly cultivated. Thus, the integration of advanced IR Biotyper applications into microbiological laboratory routines could have the potential to improve hospital hygiene practices in the future as the rapid initial delineation of nosocomial outbreaks would enable the early, focused implementation of countermeasures.

## Data Availability

The data presented in the study are deposited in the Figshare repository, https://doi.org/10.6084/m9.figshare.30999076.
